# Mood induction alters attention toward negative-positive stimulus pairs in sheep

**DOI:** 10.1038/s41598-019-44330-z

**Published:** 2019-05-23

**Authors:** Camille M. C. Raoult, Lorenz Gygax

**Affiliations:** 1 Centre for Proper Housing of Ruminants and Pigs, Federal Food Safety and Veterinary Office FSVO, Agroscope, Tänikon 1, CH-8356 Ettenhausen, Switzerland; 2 Animal Welfare Division, Veterinary Public Health Institute, Vetsuisse Faculty, University of Bern, Länggassstrasse 120, CH-3012 Bern, Switzerland; 3 Animal Husbandry & Ethology, Albrecht Daniel Thaer-Institute of Agricultural and Horticultural Sciences, Humboldt-Universität zu Berlin, Unter den Linden 6, 10115 Berlin, Germany

**Keywords:** Psychology, Animal behaviour

## Abstract

Mood is a lasting affective state that influences motivation and decision-making by pre-shaping a subject’s expectations (pessimism/optimism). Mood states affect biases in judgment, memory, and attention. Due to a lack of verbal report, assessing mood in non-human animals is challenging and is often compromised by intense training sessions. Measuring mood using attentional biases can circumvent this problem, as it takes advantage of observing a spontaneous reaction. As in humans, we expected that negative mood will heighten attention toward negative compared to positive stimuli. Here, we validate measures of attention toward acoustic stimuli in sheep (N = 64) and assess sheep’s differential attention toward acoustic stimuli before and after mood induction (N = 32). Mood was induced by manipulating the environment. We used animal vocalizations (dog barking and sheep bleating as negative and positive stimuli, respectively) varying in intensity and played simultaneously from one side each, and measured lateral attention based on the sheep’s behavior. Overall results were somewhat ambiguous. Yet, negative mood sheep seemed to shift their attention more toward dog vocalizations when the stimulus pair was well balanced at baseline. Though some adaptations are still needed, our approach could be a promising alternative to measure animals’ mood without prior training.

## Introduction

Mood is a lasting affective state that influences motivation, learning and decision-making. Mood shifts thresholds and modulates at which critical value of a stimulus an action is taken by a subject^1–3^. Particularly in situations of ambiguity and uncertainty, the expectations of a subject, i.e. its relative pessimism or optimism, is related to its negative or positive mood state^3–5^, respectively. Contrary to emotions which are intense and short-lived affective reactions to particular events, recent findings suggest that mood is a more diffuse state and depends on cumulative mismatches, i.e. it reflects the cumulative impact of repeated situations where outcomes did not coincide with expectations^1^. Negative mood may taint all experiences, at least in human clinical depression^6–8^. On the other hand, it seems that subjects in a more positive mood state deal more easily with negative short-term experiences^4,9^. Accordingly, mood is highly relevant for animal welfare and essential for the basic understanding of animal decision-making.

Because animals are non-verbal, other approaches than the questionnaire-based methods applied in humans are needed to infer mood states in animals^10^. The cognitive judgement bias test has been introduced as the standard approach to assess mood in animals^4,5,11^. This test consists of training subjects to associate two cues on the same physical axis with a negative and a positive outcome. The reinforcers in this training often consist of food rewards and, therefore, the animals’ reaction toward the cues is influenced by the motivation to reach food. Subjects are then confronted with an ambiguous intermediate cue and their behavioral reaction is observed. Based on that reaction, it can be assessed whether the subjects judged the ambiguous cue as being more similar to the negative or the positive cue and hence being associated with the negative or positive outcome (pessimistic or optimistic reaction, respectively). As this approach relies on learnt responses to relatively arbitrary and novel stimuli, the meaning of the animals’ reaction toward ambiguous stimuli is not strongly influenced by previous experiences with these stimuli. However, this test has limitations such as the necessary intense and long training of the animals which needs a lot of time and effort^12^. In addition, the training itself may interfere with the mood state of the subjects being tested^5,13,14^. Attempts at alternative approaches have been made^15–19^, but these may still not be so easily applicable and are not testing a bias in a narrow sense (i.e. both positive and negative stimuli were not presented simultaneously so the animals could not indicate their preferential attention directly).

In cognitive bias studies, an attempt has usually been made to manipulate the mood state of the animals (so called “mood induction”^5,20^). Mood can be experimentally induced using either one or multiple short-term environmental changes, long-term modifications or psychoactive drugs. The use of various types of environmental manipulations (e.g. shearing, isolation, contact with a predator, and food reward, brushing, novel objects) to induce negative and positive mood is known to be efficient^5,21^ in sheep, and cognitive biases in the direction predicted have been found using these methods^13,22–25^.

The tendency to pay biased attention toward a given stimulus within a selection of stimuli is also affected by mood^10^. Observing an animal’s attention takes advantage of a spontaneous reaction. For this, animals may need to be trained far less compared with the cognitive bias test, which requires an operant reaction. Attention is often directed to stimuli at some distance. Therefore, the visual and auditory sensory channels may be specifically used to trigger attention. For example, subjects typically gaze toward objects or details within a scene that are of interest. In several studies, this interest was found to be heightened based on the potential of the details in the scenes to trigger an emotional reaction^26^. Moreover, it is thought that attention to negative or positive details can be biased by a subject’s mood state (e.g. attracting a larger amount of attention). In humans, anxiety leads to attention biases toward threat^27,28^. More generally, negative mood leads to heightened attention toward negative compared with positive stimuli^29,30^. In sheep^16,17^ and cows^15^, animals treated with an anxiogenic drug attended the threat of a dog longer than control animals which, in turn, seemed to show more pronounced attention toward the dog than animals treated with an anxiolytic drug. This was interpreted as an attention bias, though, in these studies, the experimental animals could not choose between a negative and a positive stimulus, thus could not indicate their preferential attention. In a more recent study, Monk, *et al*.^18^ confronted sheep first to a real dog through a window for 3 s, and then to a sheep stimulus (photographs or 3D model). They found that drug induced depressive-like sheep paid more attention toward the threat (empty window) and less toward the sheep stimulus, while drug induced anxious-like sheep showed a reversed attention bias. The two stimuli were of different quality here (real dog, picture of sheep) and were not presented simultaneously. Therefore this paradigm may not reflect an attention bias in a narrow sense. Studying mood could be further simplified by using only standardized stimuli instead of real animals. To measure the differential attention animals show to two stimuli presented simultaneously, Bethell, *et al*.^19^ presented rhesus macaques with image pairs of aggressive and neutral faces of conspecifics, and found attention biases. Following a health check by a veterinarian (a presumably negative experience), the macaques’ initial vigilance toward the aggressive face changed quickly to a sustained avoidance of the aggressive face, while, following a period of enrichment (a presumably positive experience), they showed a sustained attention toward the aggressive face. These findings highlight a potential issue when interpreting attention. Attention toward a stimulus can have two distinct meanings, either curiosity (e.g. for novelty) or vigilance (e.g. toward a threat), while no attention toward a stimulus can reflect either little interest or active avoidance. However, the subjects’ body language (e.g. active vs. freezing behavior, head higher up and passive ears vs. head down or shaking) could help to disentangle which type of response is observed. Moreover, animals might differ in their previous experience with the stimuli used and, therefore, a given stimulus may vary in its emotional content among different individuals.

Visual stimuli have been used in sheep to study their recognition skills^31,32^. However, applying video stimuli projected simultaneously proved to be difficult in measuring attention in a previous experiment^33^, because we could not identify where sheep were looking and they may not perceive video images designed for human vision well. Playbacks of acoustic stimuli could present an alternative, as hearing in sheep differs less from humans in comparison with vision. It is thought that auditory attention in sheep could be assessed by tracking head and ear movements and positions^34^. Though with our previous work^33^ we were not able to measure differential visual attention when tracking sheep’s head and ears positions, such indicators might be more meaningful when investigating auditory attention, specifically when sound sources are far apart.

Dogs and humans seem to represent some form of threat for sheep (negative valence^16,35–38^), while conspecifics seem to be of positive valence^37,39^. Sheep distinguish between threatening (e.g. human, dog), and non-threatening stimuli (e.g. cardboard box, goat, sheep) in that they stay further away from an aversive stimulus, remain more vigilant, and explore it less^36,40^. When confronted with a real dog, sheep show more fearfulness^36^, higher adrenocortical responses^41^, higher heart rates^38,42^, freezing behavior, and vigilance toward the stimulus^16,17^. On the contrary, in the presence of a sheep picture, sheep show less fearfulness^39^, and approach the stimulus quickly^40^. It can thus be concluded that dog stimuli are experienced as relatively more negative and conspecifics as positive by sheep. Such stimuli may therefore provide a good pair of stimuli assessing attention bias. If a negative and positive stimulus were presented simultaneously, the differential attention that is paid to them could be measured directly. During the presentation, the stimuli should be combined in a balanced way. If we want to measure a shift in attention toward either the negative or the positive stimulus, the attention toward these stimuli should be close to equal at baseline. Only then, we can expect to be able to detect a shift towards one or the other stimulus.

The aim of our study was to validate measures of attention toward acoustic stimuli (white noise, dog and sheep vocalizations) in two groups of sheep (habituated or naïve), to find additional support for the valence of the acoustic stimuli used, and to assess differential attention toward these stimuli pre and post negative and positive mood induction (attention bias). In the validation part, we established an ethogram allowing to quantify the attention sheep directed toward acoustic stimuli played from one (white noise) or two (animal vocalizations) sides of a sheep. We used dog vocalizations (at two intensities mimicking two distances) as presumed negative and sheep vocalizations (at two intensities) as presumed positive stimuli. We predicted that sheep would turn their head toward the side where white noise was presented. Moreover, we expected that sheep would pay attention preferably to more salient stimuli when dog and sheep vocalizations were presented simultaneously, i.e. to relatively closer auditory stimuli (i.e. louder stimuli) especially when they were negative (i.e. dog vocalizations). Finally, we wanted to measure affect-driven shifts in attention in sheep after mood induction (attention bias test). We hypothesized that sheep in a more negative mood would shift their attention more toward negative acoustic stimuli compared to sheep in a more positive mood, specifically if the stimulus pair was well balanced at baseline.

## Results

### Validation experiment: sheep pay attention toward acoustic stimuli

**Figure 1:**
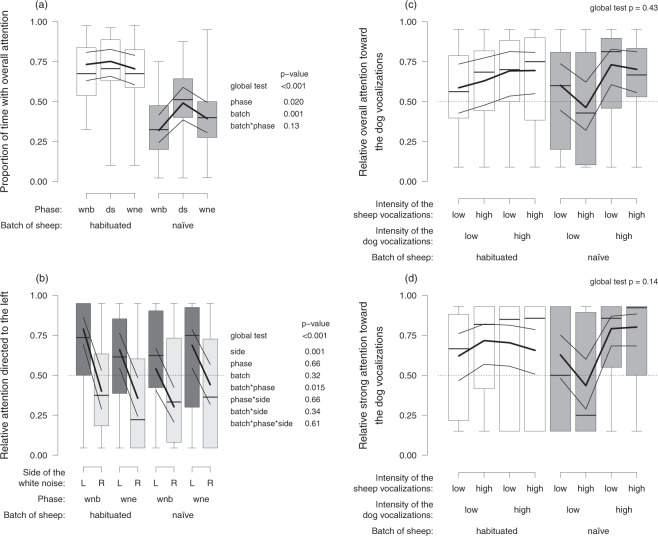
Sheep pay attention toward acoustic stimuli. (**a**) Proportion of time with sheep’s overall attention during the different phases, i.e. toward the white noise (wnb beginning, wne end) and the dog/sheep vocalizations (ds) depending on the batch of sheep (habituated, naïve). (**b**) Relative attention directed to the left when the white noise was played from the left or right side (L, R) for each phase of the white noise presentation (wnb beginning, wne end) and the batch of sheep (habituated, naïve). (**c**) Relative overall attention and (**d**) relative strong attention directed toward the dog vocalizations depending on the intensity of the sheep and dog vocalizations (low, high) and the batch of sheep (habituated, naïve). Statistical information is given for each model. Boxplots indicate data range, median, as well as lower and upper quartiles. Thick black lines are the model estimates, and thin black lines are the 95% confidence intervals of the maximum model (including the main effects and interactions).

Habituated sheep showed a higher proportion of overall attention (duration of being attentive divided by the total duration of all stimuli presented) than naïve sheep. Additionally, sheep were more attentive during the dog/sheep vocalizations than the white noise trials (Fig. 1a).

Sheep focused their relative attention more toward the side where the white noise was played regardless of the phase within a session (relative attention to the left: duration of attention to the left divided by the total duration of attention for a given stimulus; Fig. 1b). Habituated sheep directed their attention more toward the left at the beginning of the session while naïve sheep did so more at the end of the session (Fig. 1b).

During trials with animal vocalizations, sheep showed a higher relative overall attention (duration of attention toward the dog divided by the total duration of attention for a given stimulus) toward high intensity dog vocalizations. However, this effect was not supported statistically (global p-value; Fig. 1c). When looking at the relative strong attention only (duration of strong attention toward the dog divided by the total duration of strong attention for a given stimulus), the same qualitative but slightly stronger pattern could be observed for the naïve sheep only (Fig. 1d). Habituated sheep seem to show a higher relative strong attention toward the dog vocalizations, irrespectively of the intensity of the dog/sheep stimuli (Fig. 1d).

Naïve sheep vocalized more than habituated sheep (median [lower; upper quartile]: 14.5 [6.75; 20.25] vs. 0 [0; 1] times per session, respectively; W = 855.5, p < 0.001).

### Attention bias test: negative mood induction leads to an attention bias

Overall, sheep were again more attentive during the dog/sheep vocalizations than the white noise trials (phase, p = 0.019) independently of the mood group (mood, p = 0.75) and time-point (pre/post mood induction, p = 0.49; see Supplementary Fig. S2).

We found that sheep focused their attention more toward the side where the white noise was played (side, p = 0.003) regardless of the phase (phase, p = 0.17), mood group (mood, p = 0.16) and time-point (pre/post mood induction, p = 0.83), albeit the mood group and time-point modulated attention to some extent (interaction: side x mood x time-point, p = 0.026; see Supplementary Fig. S2).

**Figure 2:**
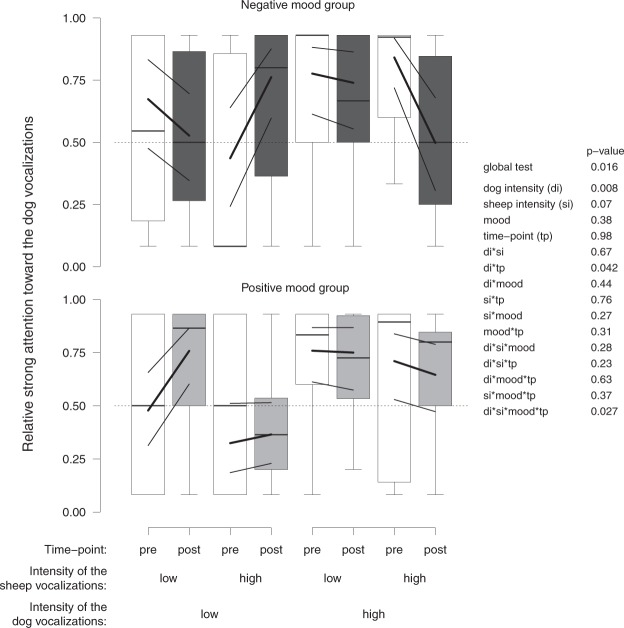
Changes in strong attention from pre to post mood induction. Relative strong attention directed to the dog vocalizations depending on the intensity of the sheep and dog vocalizations (low, high), the mood group (negative, positive), and the time-point (pre/post mood induction). Statistical information is given. Boxplots indicate data range, median, as well as lower and upper quartiles. The white, dark-grey and light-grey boxplots represent the attention pre mood induction, post negative mood induction and post positive mood induction, respectively. Thick black lines are the model estimates, and thin black lines are the 95% confidence intervals of the maximum model (including the main effects and interactions).

Concerning the animal vocalizations trials, we found a similar qualitative pattern for the relative overall attention and the strong attention, but it was not supported statistically for the overall attention (see Supplementary Fig. S3). Pre mood induction, sheep showed more strong attention toward the dog vocalizations when the dog vocalizations were of high intensity independent of the intensity of the sheep vocalizations and the subsequent mood group (Fig. 2, white boxplots). Post mood induction, changes in this attention pattern were visible. Positive mood sheep shifted their strong attention toward the dog when both the dog and sheep vocalizations were of low intensity (Fig. 2, bottom). Negative mood sheep shifted their strong attention toward the dog when the dog vocalizations were of low intensity and the sheep vocalizations of high intensity (Fig. 2, top) and shifted their strong attention toward the sheep when the dog and sheep vocalizations were at the same intensity (Fig. 2, top).

Negative mood sheep vocalized more post than pre mood induction (median [lower; upper quartile]: 19 [14.75; 21.25] vs. 10 [6.75; 15.75] times per session, respectively), while positive mood sheep vocalized less post than pre mood induction (median [lower; upper quartile]: 11 [8.5; 22] vs. 17 [14.5; 25] times per session, respectively). Yet, this pattern was not strongly supported statistically (global p-value: 0.061).

For most outcome variables, the variability within the sheep was clearly larger than between sheep indicating that the moment-to-moment reaction toward the stimuli used led to larger variability than the individual-specific way of reacting (see Supplementary Table S2).

## Discussion

Here, we established an ethogram allowing to quantify differential attention toward acoustic stimuli in sheep. In addition, the results indicate that dog barking is more salient than sheep bleating supporting the presumed valence of the stimuli, though this pattern did not reach a low p-value in the validation experiment. Though the results were ambiguous to some extent, it seems that sheep in a more negative mood shifted their strong attention toward dog vocalizations when the stimulus pair was well balanced at baseline. This specific change reflected the expected change in attention bias, though other changes were more difficult to interpret.

In our validation experiment, sheep of both batches consistently switched their relative attention to the side from where the white noise was played and did so persistently even at the end of their experimental sessions. This indicates that our ethogram is valid in reflecting to which side acoustic attention is paid in sheep. Moreover, sheep paid relatively more attention to the animal vocalizations than the white noise, which is coherent with the notion that animal vocalizations are more salient. The sheep’s reaction toward the dog and sheep vocalizations was less clear. Sheep seemed to adjust their attention to the type of call and the intensity of the stimuli: sheep generally focused their attention toward the dog vocalizations, at least when the dog vocalizations were of high intensity. This pattern was clearer in the naïve sheep and was not consistent enough across both batches to yield a low p-value. Nevertheless, this qualitative pattern is consistent with the assumption that dog vocalizations were more negative than the vocalizations of conspecifics. As prey animals, it is meaningful for sheep to be more attentive to threatening stimuli (i.e. dog vocalizations). Previous studies established that real dogs and recording of dog barking are aversive for sheep^41,42^. In humans, Arnaudova, *et al*.^43^ found that attention was captured preferentially by signals of increasing threat imminence. In our case, this would correspond to the situation when dogs are barking at a closer simulated distance. Habituated sheep were more attentive in general and vocalized less than naïve sheep during the test. Habituation seemed to decrease the challenge of the test situation and may have helped sheep to focus on the acoustic stimuli. This is consistent with Raoult and Gygax^33^, who found that habituated sheep focused more on silent video stimuli than unhabituated sheep. Nevertheless, the naïve sheep in the present study reacted more specifically to the type and amplitude of the animal vocalizations than habituated sheep. Although the naïve sheep might have had less contact with a dog, they were younger than the habituated sheep and had not been habituated to be tested nor isolated from pen mates prior to testing. This might have made them more fearful^42,44^ and thus perceive novelty more negatively. However, their more specific reaction may not have depended on them being naïve per se but may have been the result of the changes we made in our methodological approach and the previous experience of the sheep. For the naïve sheep, we increased the difference of the two amplitudes between the bleating and barking. Moreover, the bleating stimuli were from unknown sheep, while they were from familiar sheep for the habituated sheep. Also, habituated sheep might not have reacted in the same way than naïve sheep because they had previous herding dog contact at an early age. Therefore, the choice of stimuli and the animals’ characteristics (e.g. age, previous manipulations, and past experiences with dogs) seem to have an effect on how animals react. This could make it more difficult to generalize the results of the conducted test to other populations and species. In that sense, the judgement bias test is advantageous, as the cues used are relatively arbitrary and thus not as directly affected by past experiences. Here, studies that experimentally manipulate previous experience of the animals (sheep) with the stimuli (dog vocalizations), the testing situation, and the familiarity of the sheep vocalizations may shed further light on how stimuli in future studies need to be validated before putting them to use in more practical on-farm settings.

Relying on our ability to assess sheep’s attention, we compared the naïve sheep’s attention pre and post mood induction. First, we found that overall attention was kept up by the sheep in the re-testing situation post mood induction and that they still payed somewhat more attention to the animal vocalizations than to the white noise. In general, they still switched attention to the side of the white noise stimulus but did so less clearly during re-testing. This may have been a habituation effect (see also below). Pre-mood induction, the sheep that were to undergo the positive mood treatment differentiated less clearly between the sides from where the white noise was played. Because the sheep were randomly assigned to the mood groups, this needs to be considered a spurious pattern due to randomly selecting the animals that did less clearly direct their attention toward the white noise.

The patterns in overall and strong attention toward the animal vocalizations were qualitatively similar. Yet, the patterns of strong attention were more consistent with less noise and therefore reached a lower p-value. This is why we focus on the pattern of strong attention in the remainder of this paragraph. Pre mood induction, sheep directed their strong attention more toward the dog high intensity vocalizations when dog and sheep vocalizations were played simultaneously. Post mood induction, we found differences in the attention between the two mood groups. Contrary to our hypotheses, there was no general effect that negative and positive mood sheep would have directed their attention to the dog stimulus more and less, respectively. Negative mood sheep shifted their strong attention toward the dog when the dog vocalizations were of low intensity and the sheep vocalizations of high intensity, but shifted their attention more toward the sheep vocalizations when the dog barking and sheep bleating were of the same intensity. Moreover, positive mood sheep shifted their attention toward the dog barking when both stimuli were of low intensity. Prior to mood induction (i.e. in the evaluation of the validation experiment), the attention of the naïve sheep was directed most strongly away from the dog vocalizations but not extremely so when the dog vocalizations were of low and the sheep vocalizations of high intensity (slightly less than 50%). Therefore, this stimulus combination was the most balanced and the most promising to picking up attention shifts toward the (negative) dog barking. Indeed, we observed a clear such shift (toward the dog vocalizations) for the negative mood group sheep in that stimulus combination (low intensity dog and high intensity sheep vocalizations). This specific shift confirms our hypothesis that negative mood sheep would shift their attention toward the negative acoustic stimuli (i.e. dog vocalizations) in stimulus pairs that are well balanced at baseline. This pattern also confirms the results observed in previous studies with humans which found threat-related attention bias in anxious individuals^27,28,45^. In rhesus macaques^19^, the affective state of a subject seemed to play also an important role in mediating its social attention. In a negative mood, the animals were initially vigilant toward and then turned away from negative stimuli (macaque aggressive faces). In sheep, Monk, *et al*.^18^ found that depression-induced individuals paid more attention toward a threat and less to a conspecific, while anxiousness-induced individuals showed the inverse pattern. Comparing our results with theirs, we would conclude that our sheep also showed a mood state that was similar to depression (even if it might have been less extreme). We differed in our measurement approach in that we did not need live animals as stimuli and in that we presented our stimuli in a truly simultaneous way. We also noticed that negative mood sheep vocalized more post- than pre- mood induction as well as more than positive mood sheep post mood induction, indicating that they were more challenged after mood induction.

In our study, there were additional effects that did not follow from or even went against our predictions. Negative mood sheep shifted their strong attention away from the dog, when both the dog and sheep vocalizations were of the same (low or high) intensity. Observing no change in the sheep’s reactions may not have been surprising here, because the response pre mood induction was already highly biased toward the dog vocalizations. Therefore, there was little room for an increased response toward the dog vocalizations post mood induction. A shift of attention away from the dog vocalizations when stimuli had the same intensity could potentially be related to habituation. A weak such effect was indeed visible in the positive mood sheep with both stimuli presented at high intensity, too. Yet, the increase in attention toward the dog in the positive mood sheep when both stimuli were of low intensity contradicts this notion of habituation and also our hypotheses. It remains currently unclear what may have caused this pattern. One explanation could be that the combination of low intensity stimuli was less salient overall, leading to different reactions in the negative and positive mood sheep. This needs to be further investigated, e.g. by more continuously varying the intensity of the stimuli. Given all the different aspects of the results, the approach developed here may be questioned in respect to how well it is suited to measure mood shifts. However, this can only be determined if additional research is conducted aiming at understanding the different changes that we have found. In respect to the effect of habituation, an experiment would be welcome in which the current experimental approach would be repeated several times without other intervention to investigate systematic changes due to repetition of the test. Alternatively, an additional control group without mood manipulation could be included in studies such as ours.

The specific pattern in attention shift that we have found for the stimulus combination that had been well balanced at baseline is consistent with the notion that negative mood sheep were indeed in a more negative mood as positive mood sheep. In this, we could replicate prior studies that used similar mood induction procedures and investigated this difference using cognitive judgement bias testing^13,22–25,46^. The positive mood induction also seemed to have worked because sheep showed a similar attention pattern post- than pre- mood induction even though they first underwent a negative mood treatment. Based on our positive mood treatment, they seemed to have mostly recovered to the initial state even if they may not have surpassed baseline. All in all, we found that mood induction altered sheep’s attention, which provides, to our knowledge, the first evidence of affect-driven changes in attention bias in a narrow sense in a non-primate species.

Pre-recorded animal vocalizations seem to be salient cues to test attention in sheep. Therefore, the reliance on live animals as stimuli does not seem to be necessary. Nonetheless, our approach could be taken even further. Negative stimuli could be chosen such that they are not part of the normal on-farm situation and, therefore, less sensitive to different management procedures or the animals’ past experience. Such stimuli could be e.g. howling by wolves or coyotes or arbitrary but irritating sounds such as pink noise. Alternatively, species-specific vocalizations indicating threats in agonistic encounters could potentially be used and would allow the extension of our procedure to non-prey species. The study design would thus become more generalizable using such approaches. In such a testing procedure, we would recommend to focus on strong attention because this seems to differentiate more clearly in respect to where attention is directed. We propose to include a stimulus-pair with a weak negative and strong positive stimulus, such that the animal’s attention is balanced toward the two stimuli, to assess shifts toward negative mood in future studies, corresponding to the low amplitude dog and high amplitude sheep vocalizations in our current study. Based on our results, it remains unclear how to assess shifts caused by positive mood. In principle, a stimulus pair that is well-balanced at baseline should also create the possibility for shifts away from the negative and toward the positive stimulus. More research is therefore needed to determine whether the current attention bias test would be suitable to measure both negative and positive mood shifts. The current work presents an encouraging faster and simpler method to assess mood in sheep compared to typical judgement bias tests or attention tests using stimuli involving live animals. It has the advantages of not requiring prior training of the animals and not relying on food rewards. Therefore, it might be applied with some adaptation in less experimental settings such as on-farm. Nevertheless, the current findings also highlight critical issues regarding the interpretation of the behavior of the animals in the test and the stimuli used. Therefore, the attention bias test presented here should be further developed in future studies, to improve the design (i.e. the stimuli) according to our suggestions and to validate it as an alternative to the cognitive judgement bias test.

## Conclusion

This study confirms that differential attention toward acoustic stimuli can be quantified in sheep. Though some of the results remain difficult to interpret for the moment and further research is needed, it seems that sheep in a more negative mood shift their attention toward the negative dog vocalizations when the stimulus pair is salient and well balanced at baseline. The attention bias test developed in this study may, if developed further, become a promising alternative approach to measure animals’ mood quickly and without prior training.

## Methods

### Ethical note

Animal care and all experimental procedures were carried out in accordance with the guidelines for the treatment of animals in behavioral research and teaching of the Association for the Study of Animal Behaviour (ASAB, 2012) and the current laws of Switzerland. This study was approved by the Research Commission of the Federal Food Safety and Veterinary Office, and the necessary authorisation to conduct animal experiments was granted by the cantonal authorities (Canton of Thurgau permit no. 27508-TG01/16).

### Animals

**Figure 3:**
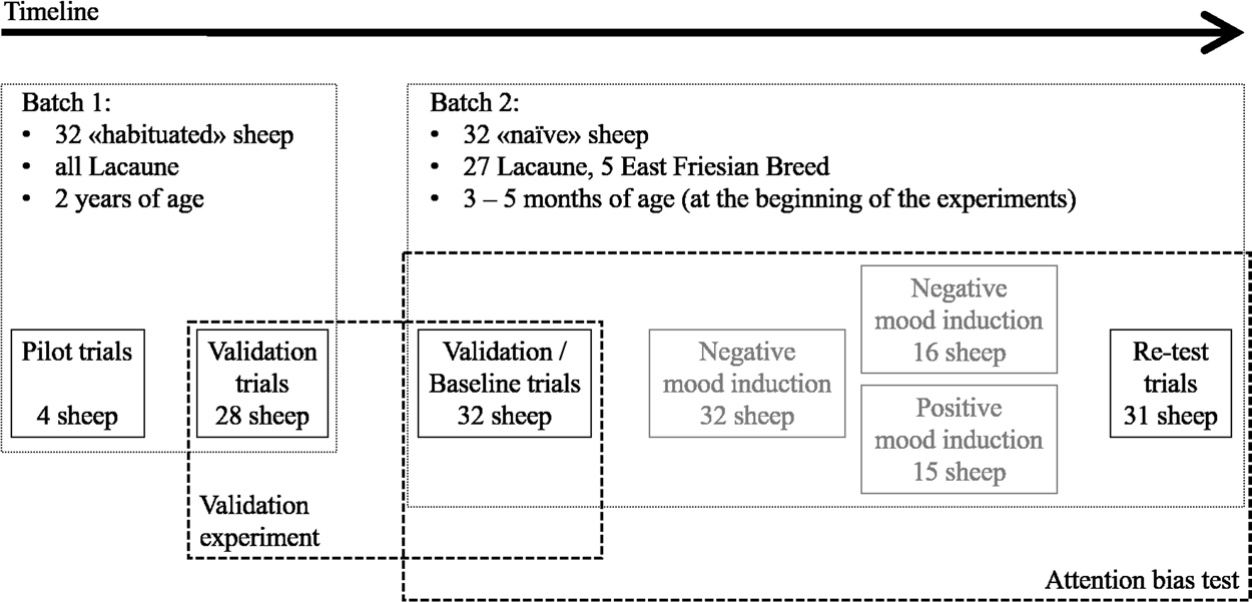
Scheme presenting the number of animals in the different steps of the study, the sequence of the different types of trials and the combinations of trials used in the evaluation.

We used a total of 64 non-lactating and non-reproducing female sheep in two batches (Fig. 3). The two batches of sheep were housed successively in eight group pens (2.4 m × 3.5 m per group of four sheep) in an open-front barn at the Agroscope Research Station in Tänikon, Switzerland. They had straw bedding available, hay was provided twice a day at regular times and water was available ad libitum. Sheep of batch 1 (referred to as “habituated sheep”) had been involved in a series of tests with visual stimuli before^33^ and might have had previous contact with the farmer’s herding dogs in their early life (at an age younger than 5 months), while sheep of batch 2 (referred to as “naïve sheep”) were naïve in respect to being tested and had, to our knowledge, no previous contact with a dog. We used the habituated and naïve sheep to validate our approach to measure attention. With the habituated sheep, a pilot trial was conducted using the four sheep of one randomly chosen group, in order to establish an ethogram aiming at measuring to which side a sheep directs its attention and have a general idea of the sheep’s reactions toward the different stimuli (see also Supplementary Methods). These sheep were no longer used in the formal experiment. Therefore, 28 habituated and 32 naïve sheep were tested in the validation trials (Fig. 3). Before testing, all the animals were habituated to be handled by the same experimenter (CR). Each group of four sheep was then familiarized with a mobile pen (2.3 m × 2 m wooden structure without floor and mounted on wheels), used to move the sheep from the home pens to the test arena. The validation experiments took place on March 14^th^ (habituated sheep) and May 22^th^ 2018 (naïve sheep). For one naïve sheep, the test needed to be aborted because it got stuck in the feeding station of the test set-up (see below; the incomplete session was included in the analysis, see also Supplementary Methods).

At the same time, the validation experiment served as a baseline for the naïve sheep before their mood was experimentally changed and re-assessed (i.e. the attention bias test; Fig. 3). For the mood induction, the eight groups of four naïve sheep remained housed as before (in a 2.4 m × 3.5 m pen for each group), at least during the night. Two animals of each group received the negative mood induction treatment whereas the other two received the positive mood treatment (see also *Mood induction procedure)*. The attention bias test was re-applied on the day after the last mood induction day with the naïve sheep only. Only 31 sheep were available for re-testing because one sheep (East Friesian, positive mood group) had died in the meantime due to a cause unrelated to the current experiment. The attention bias test post mood induction took place on July 2^nd^ 2018.

### Experimental procedure

**Figure 4:**
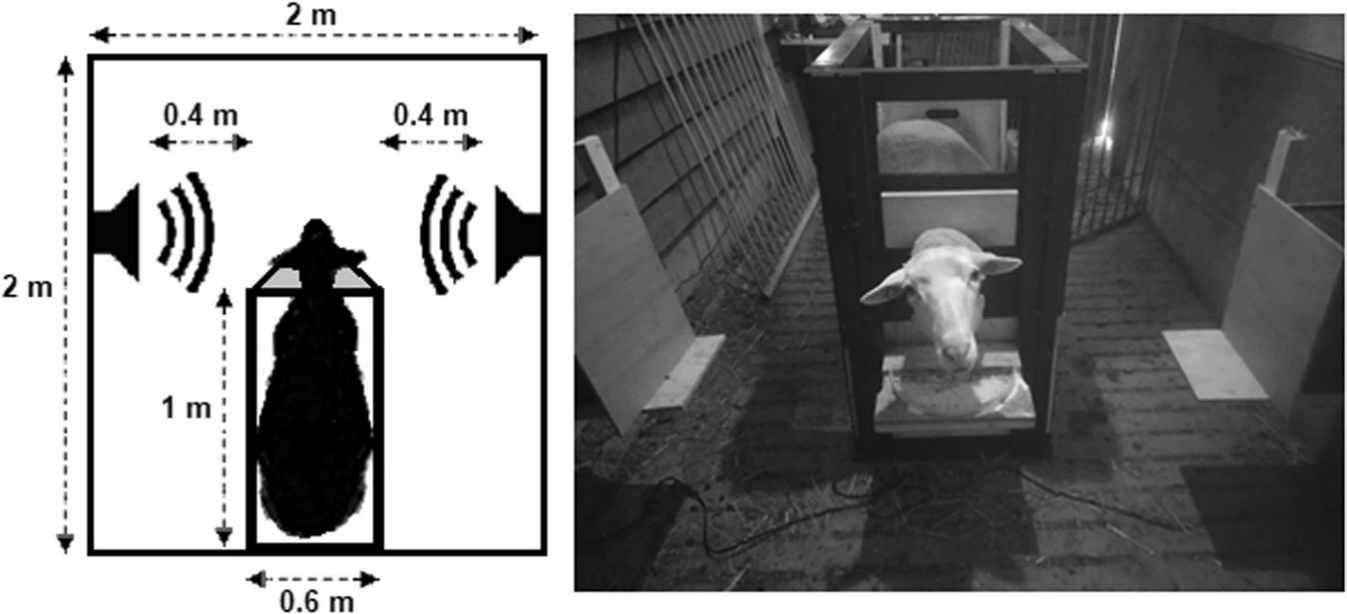
Scheme (top-view; left) and photograph (right) of a sheep in the feeding station used for the presentation of the acoustic stimuli.

In the validation experiment as well as the attention bias test, the sheep were individually tested (without prior training for the naïve sheep) while being restricted in a feeding station (Fig. 4). This station consisted of a box of the size of a single animal (1 m × 0.6 m). It had wire mesh walls on both sides and a trough at its narrow end. Sheep were restricted in their movement by the walls of the feeding station while their head was blocked by a sliding board over their neck preventing them from removing their head from the trough (Fig. 4). To encourage the sheep to go into the station, a small amount of food (a mixture of UFA 763 ProRumin COMBI QM, Herzogenbuchsee, Switzerland) was available in the trough at the start of each session. The trough opened to a space in which two loudspeakers (Edifier S2000V Multimedia speaker, Beijing Edifier Technology Co., Ltd., China) were positioned at an equal distance (40 cm) on the right and left side of the sheep’s head (Fig. 4).

During the test, the sheep did not have any visual or auditory contact with the other sheep and had no human experimenter in view. Whereas the habituated sheep had gone through similar test set-ups before, the separation from pen mates was novel for the naïve sheep. Each sheep underwent one session for the validation experiment lasting 2 min 10 s and remained in the feeding station for the entire duration. The same type of session was used in the naïve sheep in the attention bias test after mood had been influenced experimentally. The session was composed of 8 trials of 10 s each with inter-trial durations varying randomly between 5 and 10 s. Feed in the trough was usually eaten before or within the first few trials. The first stimulus started approximately 30 s after the sheep’s head was blocked in the feeding station. The session started with 2 trials during which a white noise was played once on the right and once on the left side. The side where the white noise was first played was balanced between sheep. The next 4 trials consisted of playbacks of dog vocalizations from one side and sheep vocalizations played simultaneously from the other side. The session ended with another 2 white noise trials, once from each side. The side from which the white noise was first played was again balanced across sheep (see also Supplementary Methods for a detailed description of the stimuli and stimuli preparation). The four stimuli including animal vocalizations were manipulated by varying the side from which the stimuli were alternatingly being played, and the intensity (i.e. two amplitudes mimicking two different distances) of the two stimuli (i.e. four possible combinations; see Supplementary Methods). The sequence of these four stimuli was balanced between sheep. Any stimulus including vocalizations was played to a sheep only once to avoid habituation. Each tested sheep at each time point had its own sequence composed of two times the white noise, the four possible animal vocalizations combinations, and another two times the white noise.

Based on the white noise trials, we wanted to show that we can assess to which side the attention of the sheep was directed and whether it changed throughout the session. We expected that sheep would direct their attention towards the speaker broadcasting the white noise. If there was habituation within the session, the specificity of this reaction might decline, i.e. sheep might not differentiate the sides as clearly at the end of the session compared with the beginning. With the trials including animal vocalizations, we wanted to show potential shifts in attention due to the type and distance of the stimuli. We expected that sheep would direct their attention to the dog if the dog vocalizations were loud and when the dog vocalizations were of the same intensity as the sheep vocalizations. This was expected to be more balanced with low intensity dog vocalizations and high intensity sheep vocalizations. Before the attention bias test, we induced a negative (n = 16) and a positive (n = 15) mood state in the naïve sheep. With that, we wanted to show that affect-driven attention biases occur, in particular, attention of the sheep in a more negative mood shifting more toward the dog vocalizations. We expected to see this specifically well in the case of low intensity dog vocalizations and high intensity sheep vocalizations because of the relative similarity in the attention elicited at baseline.

### Mood induction procedure

For the attention bias test, we induced negative and positive mood states in sheep that had been shown to result in mood differences in earlier cognitive bias studies^13,22–25,46^. We applied short-term changes in the environment of the sheep, i.e. deteriorating or improving conditions repeatedly to induce cumulative mismatches. Because we assumed that our sheep were in a rather positive mood in their housing environment during the validation phase of the study (e.g. conditions were better than Swiss minimal standards), and to induce a contrast, all sheep were first treated as to induce a negative mood. We exposed all the 31 naïve sheep to various unpredictable, uncontrollable and aversive events, occurring at different times of the day and for various durations over a time period of 2 weeks (see Supplementary Table S3a,c). These events were based on husbandry procedures common to production systems. Over another time period of 2 weeks, we continued to expose half of the sheep (total n = 16) to further aversive events, to maintain a negative mood, while we exposed the other half of the sheep (n = 15) to repeated predictable positive events (Fig. 3; see also Supplementary Table S3b,c), to induce a positive mood. To do so, half of the sheep in each housing group were randomly assigned to either the negative or positive treatment (but no sheep stayed alone in the home pen as the single sheep was assigned to the positive treatment and was thus with other conspecifics at any time).

To facilitate sheep associating different types of human handling, experimenters wore all-white clothing during aversive events, normal work clothes (blue or green) during positive handling (i.e. habituation to the mobile pen, at feeding times and throughout other positive events), and white clothes with horizontal blue stripes during the attention bias test after mood induction as in^22,24^.

On the day following directly the four weeks of mood induction, all the 31 sheep were re-tested once, as previously for the validation (see *Experimental procedure*).

### Behavioral measurements

Head and ear positions and movements were recorded using a video camera (camcorder Sony DCR-SX33E, Sony Corporation, Tokyo, Japan). We developed an ethogram to measure sheep’s attention, i.e. whether the sheep was attentive or not, and recorded the side to which the attention was directed, and how strongly sheep did so throughout the complete test sessions (stimuli and inter-stimuli intervals; see Supplementary Methods, Supplementary Table S1, and Supplementary Fig. S1).

The number of sheep vocalizations for each sheep and session was also counted from the video recording as an indicator of general arousal.

### Statistical analysis

Statistical analyses were performed in R version 3.5.1^47^. All the attention outcome variables (see Supplementary Table S1) were expressed as proportion of time (that sheep were attentive) during the different phases of a session or the presentation of a single stimulus and logit transformed. They were then used as continuous outcome variables in linear mixed-effects models (R package lme4^48^) to correctly account for dependencies in the data. Statistical assumptions were checked using graphical analysis of residuals focusing on the distribution of errors and random effects, and homoscedasticity of errors of the models (R package DHARMa^49^). We followed a full model approach, i.e. we set up a maximum model that we present and interpret^50^. First, we calculated the global p-value (between the maximum and null model) using parametric bootstraps (R package pbkrtest^51^). If that model reached a low p-value, we tested each of the predictor variables singly by comparing the full model to the one omitting this predictor. In order to allow for meaningful comparisons for main effects when interactions formed part of the model, we used sum-contrasts for our predictor variables. Model predictions were calculated based on semi-parametric bootstraps for mixed models (R package boot^52^). The specific models (i.e. the included fixed and random effects) set up for the validation experiment and attention bias test can be found in Supplementary Methods.

The “overall (directed) attention” (i.e. the sum of the weak and strong attention that was directed to either side) was summed across the white noise stimuli at the start of the test session, the dog/sheep vocalizations, and the white noise stimuli presented at the end of the test session and expressed as a proportion of the overall stimulus length in each of the three phases. This outcome variable was used to assess the change in general attentiveness throughout the test session (validation test) and additionally from baseline to post mood induction (attention bias test). All further outcome variables were expressed as the proportion of overall attention (=“relative” attention) that was directed to the left speaker (during white noise trials) or to the dog vocalizations (during animal vocalization trials; see Supplementary Table S1). The overall attention for each stimulus was used as a weight in these evaluations, such that stimulus presentations with longer attention phases were weighted more strongly. The analysis of the attention directed to the left speaker aimed at establishing that the sheep followed the white noise with their attention and did consistently so in the different phases of the experiment. The evaluation of the attention toward the dog (rather than the sheep) vocalizations aimed at establishing which stimulus pair would present a balanced baseline and how the attention changed with mood induction.

## Supplementary information

Supplementary Information

Supplementary Data S1

Supplementary Data S2

## Data Availability

All datasets and a short description of the outcome variables, fixed effects, and random effects, for the validation experiment (Supplementary Data S1), and the attention bias test (Supplementary Data S2) are publicly available with this study online at *address*.
