# Supplementary material for: Mood induction alters attention toward negative-positive stimulus pairs in sheep
Source: Sci Rep. 2019 May 23;9:7759. doi: 10.1038/s41598-019-44330-z (PMC6533262; doi:10.1038/s41598-019-44330-z)
Supplement: Supplementary file 1 — Supplementary Information [file 41598_2019_44330_MOESM1_ESM.pdf]

Supplementary Information

Mood induction alters attention toward negative-positive stimulus pairs in sheep

Camille M. C. Raoult, Lorenz Gygax

Supplementary Methods

Preparation of the acoustic stimuli

We used two sets of stimuli. We used white noise (i.e. random noise with a uniform frequency spectrum over a wide range of frequencies: 20 Hz to 20 kHz) as a stimulus, for which we assumed no emotional content that depended on innate reactions or reactions learnt throughout ontogeny (apart from its novelty and the suddenness of its presentation). The direction of attention to one or the other side from where the noise was played back was measured. We used dog vocalizations as presumed negative and sheep vocalizations as presumed positive stimuli. These stimuli were played back simultaneously to assess the relative attention of the sheep toward the two types of stimuli. The amplitude of all these stimuli when not reduced was set to the same level.

Dog vocalizations were a mixture of barks and growls recorded from large dogs (approximately 25 short barks per 10 s stimulus). Five dogs (4 males, 1 female, 3-9 years old, 58-71 cm and 30-40 kg) of different breeds (Doberman, German Shepherd and White Swiss Shepherd) were individually recorded in response to the presence of a stranger while the dogs were enclosed alone in a kennel, inside a car with the windows open, or in a house. Calls were recorded from outside the enclosures, at distances of 2 to 3 m with a directional microphone (Sennheiser MKH70, Sennheiser electronic GmbH & Co. KG, Wedemark, Germany), connected to a digital recorder (with a sampling rate of 44.1 kHz; Marantz PMD660, Marantz Professional, Cumberland, Rhode Island, United States), before being uploaded to a computer and saved in WAV format at 16-bit amplitude resolution. We used Praat v.5.0.47 DSP Package [1] to build 11 dog vocalization stimuli, i.e. dog barking and growling of equalized amplitude in direct successions from one dog at the time, and lasting 10 s each.

For the sheep vocalizations, we recorded the habituated sheep bleating while they anticipated the delivery of fresh hay and concentrates (a mixture of UFA 763 ProRumin COMBI QM, Herzogenbuchsee, Switzerland) at a time-point before the present experiment (approximately 12 long bleats per 10 s stimulus). As in Maigrot, et al. [2] and de la Torre, et al. [3], the positive valence experienced by the bleating sheep was inferred from the context, considering that food enhances fitness and triggers approach behavior. The approach behavior could also be observed while recording the vocalizations used in this experiment. Sheep calls were recorded with the same devices as the dog calls from outside the home pens, at distances of 2 to 7 m. Calls amplitude was equalized and we built 15 sheep vocalizations' stimuli, i.e. direct successions of sheep bleating, lasting 10 s each.

Each dog and sheep vocalization stimulus was manipulated to obtain two intensities, i.e. one reduced and one full, mimicking two distances of the sound source, i.e. one close and the other further away. For the habituated sheep, both the dog and sheep stimuli were used at their full amplitude for the high intensity, and the general stimulus amplitude was halved using Praat v.6.0.37 DSP Package [1] for the low intensity. However, after the habituated sheep were tested and their behavior assessed, we decided to modify the amplitudes of the

stimuli used for the naïve sheep to make the salience of the two types of stimuli even more comparable. In order to have a more similar loudness for the high intensity stimuli, the sheep stimuli were used at their full amplitude while the general dog stimuli amplitude was reduced (amplitude at 0.8) using Praat. In order to have a stronger difference between the two intensities, we adapted the method used by Naguib, et al. [4] in songbirds. The low intensity stimuli were attenuated in a frequency-dependent manner, i.e. the relative intensities of high frequencies decreased more with increasing propagation distance. To apply this strong reduction of the stimuli amplitude, we filtered animal vocalizations at 2 dB/octave using a graphic frequency filter in Cool Edit Pro 2.1 (Syntrillium Software Cooperation, Phoenix, Arizona, U.S.A.). During a test, the sheep could hear four different barking dogs. Each vocal stimulus (at low or high intensity) was used only once per sheep and approximately the same number of times across all the sheep.

Definition of sheep's attention based on behavioural measurements

Attention was defined based on the sheep's behavior (ethogram in Supplementary Tab. S1). This ethogram was developed in a pilot test involving four sheep that were subjected to stimulus sequences close to those used in the final testing. Sheep behavior might have been accompanied by sounds, e.g. a writhing sheep moved around quickly and the feet on the floor could be heard as well as the body moving along the wire mesh of the box, that help us to confirm a specific behavior. In principle, we were interested in the overall attention that was directed to either side. Nevertheless, strong attention may be a clearer sign of where attention is focused to and, therefore, strong attention was additionally evaluated (Supplementary Tab. S1).

The same experimenter (CR) first assessed sheep's attention within each half-second interval of the videos with the sound turned off to be blinded in respect of the exact time when the stimuli were played. Without the sound, the scorer had no information on the side from which the white noise or the dog/sheep vocalizations originated nor about their intensity. In addition, the full sequence of stimuli was scored without specific knowledge of the exact time of the stimuli presentation. All these aspects assured that the observer was biased in scoring as little as possible. The behavior was then re-scored directly with the sound on to ascertain specific behaviors such as writhing, sniffing, eating, and nibbling the table. We consider assessing these half-second intervals as being very close to continuous scoring of the videos and therefore also address the respective data as durations. Intra-observer agreement for sheep's attention was assessed in a total of 30 test sessions. To do so, ten sheep per habituated and naïve batch in the validation, and five sheep per mood group in the re-assessment after mood induction were randomly chosen and their attention assessed a second time 3-5 days later by the same experimenter (CR). To quantify intra-observer agreement on weak and strong attention to the left (or the dog vocalizations) and right (or to the sheep vocalizations) we used a coefficient of agreement (R package Agreement [5]). Intra-observer agreement for sheep's weak attention to the left (or to the dog vocalizations), weak attention to the right (or to the sheep vocalizations), strong attention to the left (or to the dog vocalizations) and strong attention to the right (or to the sheep vocalizations) were 0.99, 0.95, 0.98 and

0.96, as well as 0.97, 0.92, 0.95 and 0.90, for the habituated and naïve sheep pre mood induction, respectively, and 0.89, 0.94, 0.96 and 0.94 for the naïve sheep post mood induction.

S*tatistical analysis: Validation experiment*

Test sessions from n = 28 habituated and n = 32 naïve sheep were analyzed together. The outcome variables are formally defined in Supplementary Tab. S1. For the overall attention, the maximum model included the phase (factor with three levels: white noise at the beginning, sheep/dog vocalizations, white noise at the end), the batch of sheep (factor with two levels: habituated, naïve) and their interaction as fixed effects. The random effect consisted of the sheep identity nested in the housing group. With this approach, we wanted to investigate whether the general attention changed in the course of the test sessions and whether the prior experience of the sheep influenced their attention.

For the relative attention to the left (white noise), the maximum model included the phase (factor with two levels: beginning, end of the session), the side of the stimulus (factor with two levels: left, right), the batch of sheep (factor with two levels: habituated, naïve) and all their potential interactions as fixed effects. The random effects were composed of the temporal position (beginning, end) nested in sheep identity nested in the housing group. Based on this analysis, we wanted to assess whether and how much the attention was directed to the side from where the sound originated. This was mainly to validate the usefulness of our ethogram in showing to where attention is directed. Moreover, we investigated whether there was a quick habituation to the white noise from the start of the test session to the end.

For the relative attention toward the dog vocalizations, the maximum model included the intensity of the sheep stimulus (factor with two levels: weak and strong), the intensity of the dog stimulus (factor with two levels: weak and strong), the batch of sheep (factor with two levels: habituated, naïve) and all their potential interactions as fixed effects. The random effect consisted of the sheep identity nested in the housing group. Here, we wanted to see how much the intensity of the dog vocalizations and sheep vocalizations modulated the amount of attention directed at the two stimuli. The main aim was to find a well-balanced stimulus combination, in which both stimuli would draw a similar amount of attention. Only based on such a stimulus pair, it is conceivable to detect changes in attention toward either the negative or the positive stimulus.

We compared the number of vocalizations that occurred from the onset of the first stimulus until the end of the last stimulus, i.e. during the complete duration of the test, between the two batches of sheep using a Mann-Whitney-U test. Vocalizations are often taken as an indicator of general arousal and can be viewed as a measure of how much the animals were challenged in the test.

S*tatistical analysis: Attention bias test*

Test sessions from n = 32 naïve sheep pre mood induction and the same n = 31 sheep post mood induction (n = 16 negative mood and n = 15 positive mood) were analyzed. For the overall attention, the maximum model included the phase (factor with three levels: white noise at the beginning, sheep/dog vocalizations,

white noise at the end), the mood group (factor with two levels: negative, positive), the time-point (factor with two levels: pre, post mood induction) and all their potential interactions as fixed effects. The random effect consisted of the condition (pre, post mood induction) nested in sheep identity nested in the housing group. As in the validation experiment, this analysis aimed at evaluating whether there were general changes in attention either due to the repeated confrontation with the stimuli or the mood induction.

For the relative attention to the left (white noise), the maximum model included the phase (factor with two levels: beginning, end of the session), the side of the stimulus (factor with two levels: left, right), the mood group (factor with two levels: negative, positive), the time-point (factor with two levels: pre, post mood induction) and all their potential interactions as fixed effects. The random effects were composed of the temporal position (beginning, end) nested in the condition (pre, post) nested in sheep identity nested in the housing group. Based on this analysis, we wanted to check that there were no major changes in the sheep's reactions to white noise that would invalidate the ethogram in the re-testing of the sheep post mood induction.

For the relative attention toward the dog vocalizations, the maximum model included the intensity of the sheep stimulus (factor with two levels: weak and strong), the intensity of the dog stimulus (factor with two levels: weak and strong), the mood group (factor with two levels: negative, positive), the time-point (factor with two levels: pre, post mood induction) and all their potential interactions as fixed effects. The random effects were composed of the condition (pre, post) nested in sheep identity nested in the housing group. This analysis can be considered the main focus of our study. Here, we investigated how attention changed in respect to the dog and sheep vocalizations due to the mood induction. Such changes can potentially be interpreted as attention biases.

We compared the number of vocalizations (log-transformed) of the two mood groups (negative, positive) at the two time-points (pre and post mood induction) using a two-factorial linear mixed effects model (R package lme4 [6]), with the sheep identity as the only random effect.

Supplementary Table S1. Definitions of the behavioral variables reflecting (**a**) the attention's ethogram and (**b**) the definitions of the derived outcome variables used in the statistical evaluation with detailed information about the evaluable data.

(**a**)

Ethogram	Abbr.	Definition
No attention		A sheep that kept both ears passive (positioned in the transverse plane and often appearing shorter on the video image because they were hanging down), ate (head down), sniffed, played (nibbling the table), or writhed (sheep's neck and head pulled toward the box, or moved up and down quickly and repeatedly; see Supplementary Fig. S1a-S1c)
Attention		The sheep's head was kept in the horizontal plane (when the sheep's head covers the largest area in the picture) or with the muzzle higher up, and the ears were either both forward, both backward, or asymmetrical for more than 10° from the transverse plane (see Supplementary Fig. S1d-S1i)
Undirected attention		Attention with the head kept straight (within 0 and 5° to the left or right; see Supplementary Fig. S1e, S1h)
Weak attention to the left	**WAL**	Attentive behavior with the sheep's head turned slightly (from 5 to 30° from the middle; see Supplementary Fig. S1d) to the left side
Weak attention to the right	**WAR**	Attentive behavior with the sheep's head turned slightly (from 5 to 30° from the middle; see Supplementary Fig. S1f) to the right side
Strong attention to the left	**SAL**	Attentive behavior with the sheep's head turned strongly (> 30° from the middle; see Supplementary Fig. S1g) to the left side
Strong attention to the right	**SAR**	Attentive behavior with the sheep's head turned strongly (> 30° from the middle; see Supplementary Fig. S1i) to the right side
Weak attention to the dog	**WAD**	Weak attention as defined above that was directed toward the side from which the dog vocalizations were broadcasted (independent of whether that speaker was on the left or the right)
Strong attention to the dog	**SAD**	Strong attention as defined above that was directed towards the side from which the dog vocalizations were broadcasted (independent of whether that speaker was on the left or the right)

(**b**)

Outcome variables	Only attention directed to one or the other side was considered	
[Proportion of time with] overall attention	Definition:<br>**(WAL + WAR + SAL + SAR) / (total duration of all stimuli)**	
	Validation experiment:	
	E[1]: 60 animals x 3 phases (w.n. beginning, vocalizations, w.n. end) =	180
	R[2]: 1 animal with incomplete session (1 phase missing) =	179
	M[3]: none, 179 - 0 =	179
	Attention Bias Test:	
	E[1]: 32 animals x 2 time-points (pre and post mood induction) x 3 phases =	192
	R[2]: 1 animal missing post mood ind., 1 with incomplete session, 192 - 3 - 1 =	188
	M[3]: none, 188 – 0 =	188
Relative attention to the left	Definition:<br>**(WAL + SAL) / (WAL + WAR + SAL + SAR); for each stimulus**	
	Validation experiment:	
	E[1]: 60 animals x 2 stimuli (left, right) x 2 phases (beginning and end) =	240
	R[2]: 1 animal with incomplete session, 240 - 2 =	238
	M[3]: 13 trials of 11 sheep missing (1 to 3 / sheep) = 238 - 13 =	225
	Attention Bias Test:	
	E[1]: 32 animals x 2 time-points x 2 stimuli x 2 phases =	256
	R[2]: 1 animal missing post mood ind., 1 with incomplete session, 256 - 4 - 2 =	250
	M[3]: 18 trials of 13 sheep missing (1 to 3 / sheep): 250 - 18 =	232
Relative overall attention toward the dog vocalizations	Definition:<br>**(WAD + SAD) / (WAL + WAR + SAL + SAR); for each stimulus**	
	Validation experiment:	
	E[1]: 60 animals x 4 stimulus combinations =	240
	R[2]: 1 animal with incomplete session, 240 - 3 =	237
	M[3]: 8 trials of 6 sheep missing (1 to 3 / sheep), 237 - 8 =	229
	Attention Bias Test:	
	E[1]: 32 animals x 2 time-points x 4 stimulus combinations =	256
	R[2]: 1 animal missing post mood ind., 1 with incomplete session: 256 - 4 - 3 =	249
	M[3]: 9 trials of 9 sheep missing (1 / sheep): 249 - 9 =	240
Relative strong attention toward the dog vocalizations	Definition:<br>**SAD / (SAL + SAR); for each stimulus**	
	Validation experiment:	
	E[1]: 60 animals x 4 stimulus combinations =	240
	R[2]: 1 animal with incomplete session, 240 - 3 =	237
	M[3]: 57 trials of 33 sheep missing (1 to 3 / sheep, 4 in one sheep), 237 - 57 =	180
	Attention Bias Test:	
	E[1]: 32 animals x 2 time-points x 4 stimulus combinations =	256
	R[2]: 1 animal missing post mood ind., 1 with incomplete session: 256 - 4 - 3 =	249
	M[3]: 42 trials of 22 sheep missing (1 to 6 / sheep): 249 - 42 =<br>(1 sheep missing one complete time-point)	207

[1] Expected (E) number of rows given the sample size
[2] Number of rows (R) in the data table (observed trials)
[3] Number of rows evaluable due to missing data (M) in cases where no (strong) directed attention was shown in a trial and, therefore, the relative attention resulted in a division by 0

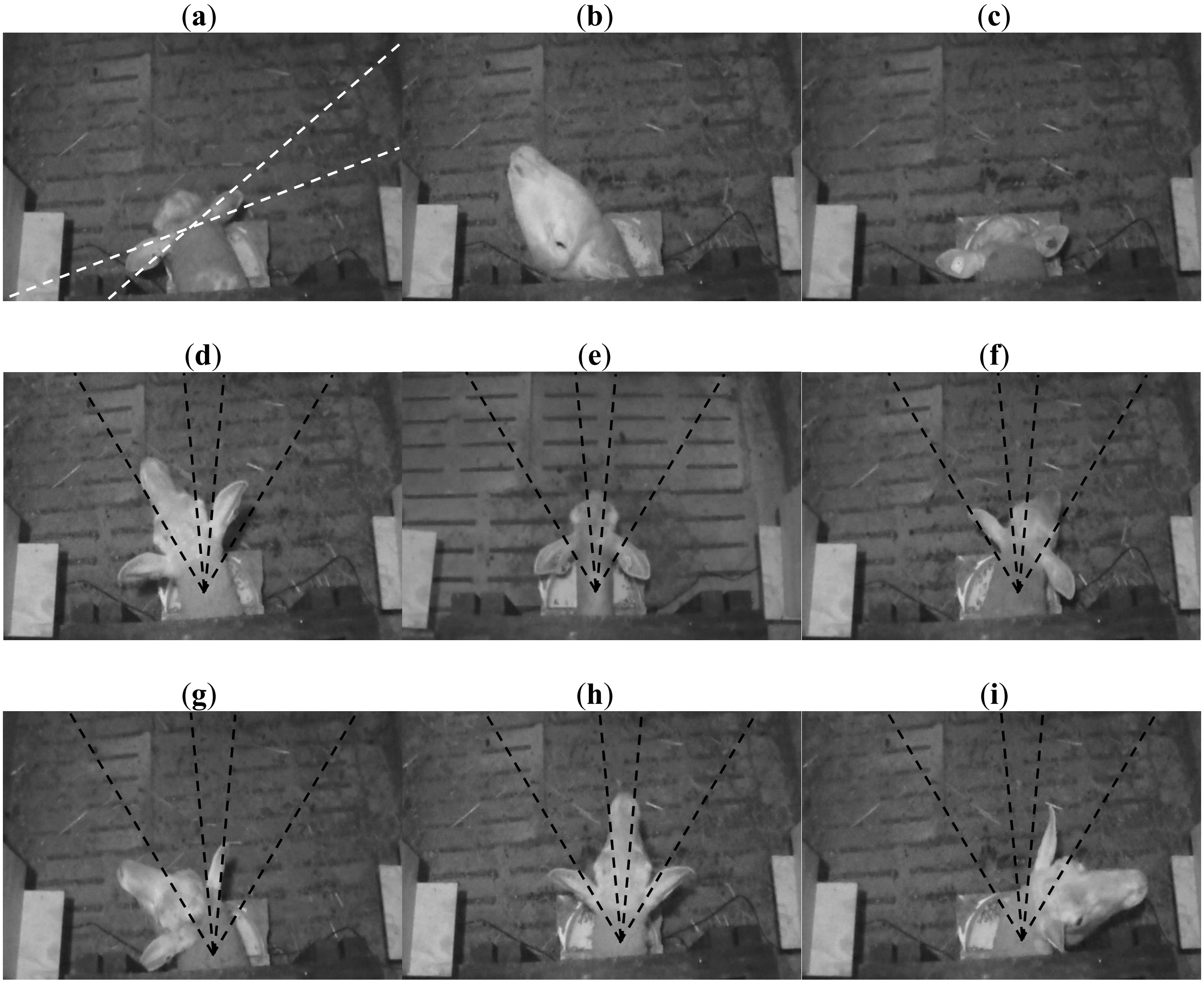

Supplementary Figure S1. Illustration of the ethogram used to define sheep's attention. Examples of non-attentive sheep: (**a**) passive ears and head down (the dotted white lines correspond to the 10° angles from the transversal plan used to define the position of the ears), (**b**) writhing, and (**c**) eating. Attentive sheep with attention directed to one side or not: (**d**) both ears forward and head slightly on the left, (**e**) both ears backward and head straight forward (no side clearly preferred), (**f**) asymmetric ears and head slightly on the right, (**g**) both ears forward and head strongly on the left, (**h**) both ears forward and head straight forward (no side clearly preferred), and (**i**) both ears forward and head strongly on the right. The dotted black lines correspond to the 5° and 30° angles used to define the side (left or right) and intensity (weak or strong) of the attention.

Supplementary Table S2. Estimated variance components of the different models; phases: beginning and end; time-point: pre and post mood induction. Exact zeros point toward numerically non-estimable effects.

Outcome variables	group-to-group variability	sheep-to-sheep variability	phase-to-phase variability	variability between time-points	Error variability
[Proportion of time with] overall attention					
Validation Experiment	0.662	0			0.706
Attention Bias Test	0	0.270		0.327	0.542
Relative attention to the left					
Validation Experiment	$2.98*10^{-16}$	0.439	0		1.360
Attention Bias Test	0	0.351	$1.565*10^{-17}$	0.052	0.916
Relative overall attention toward the dog vocalizations					
Validation Experiment	0.184	0.153			1.393
Attention Bias Test	0	0		0.001	1.082
Relative strong attention toward the dog vocalizations					
Validation Experiment	0	0.203			1.778
Attention Bias Test	$2.598*10^{-15}$	0.234		0	1.407

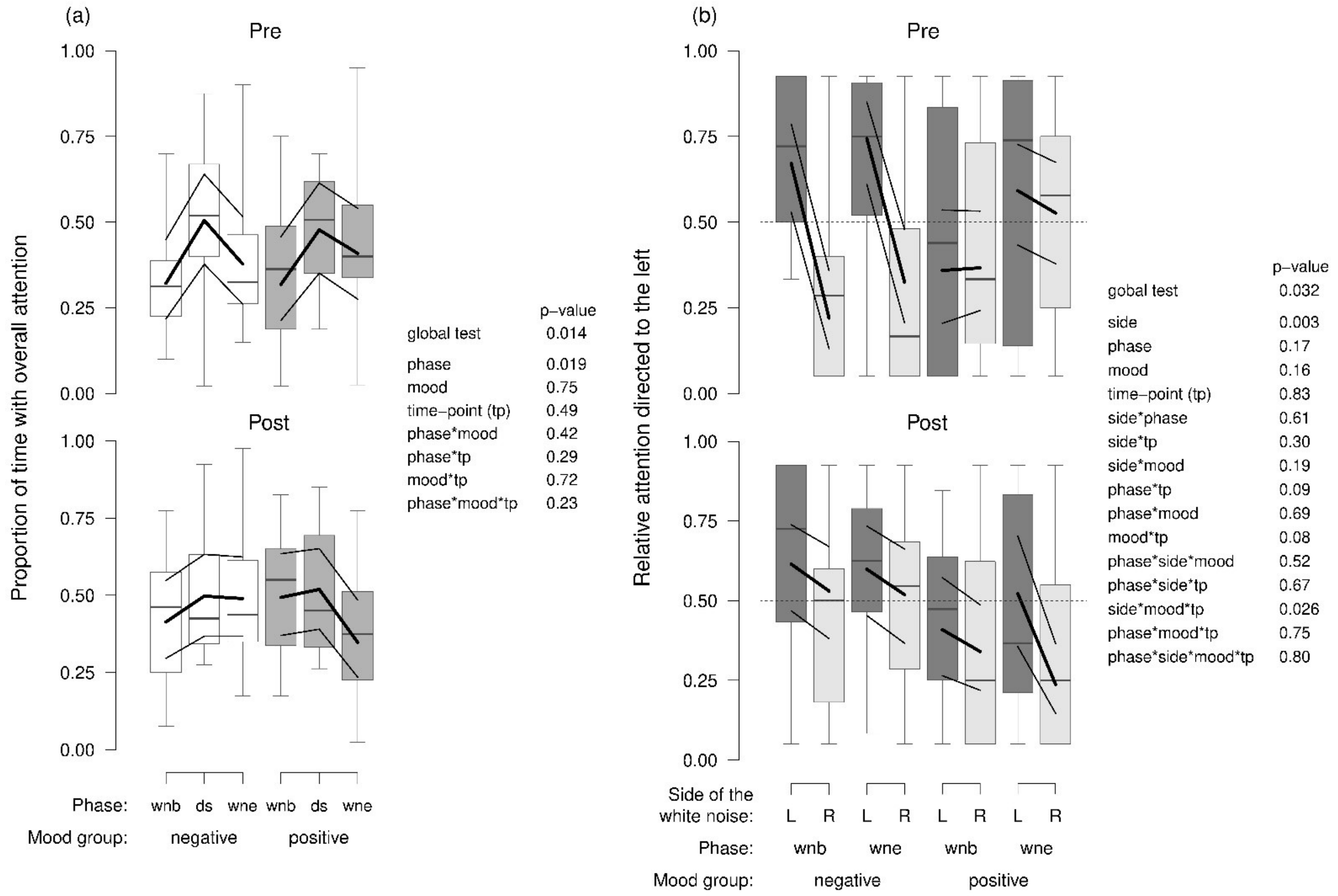

Supplementary Figure S2. Sheep still pay attention toward acoustic stimuli post mood induction. (**a**) Proportion of time with sheep's overall attention toward the white noise (wnb beginning, wne end) and the dog/sheep vocalizations (ds) depending on the mood group (negative, positive), and the time-point (pre, post mood induction). Overall, the sheep paid more attention to the animal vocalizations in comparison with the white noise. (**b**) Relative attention directed to the left when the white noise was played from the left or right side (L, R) depending on the phase of the white noise (wnb beginning, wne end), the mood group (negative, positive), and the time-point (pre, post mood induction). In general, the side from where the sound was played draw the attention of the sheep, though this was modulated to some extent by the mood group and phase. Statistical information is given for each model. Boxplots indicate data range, median, as well as lower and upper quartiles. Thick black lines are the model estimates, and thin black lines are the 95% confidence intervals of the maximum model (including the main effects and interactions).

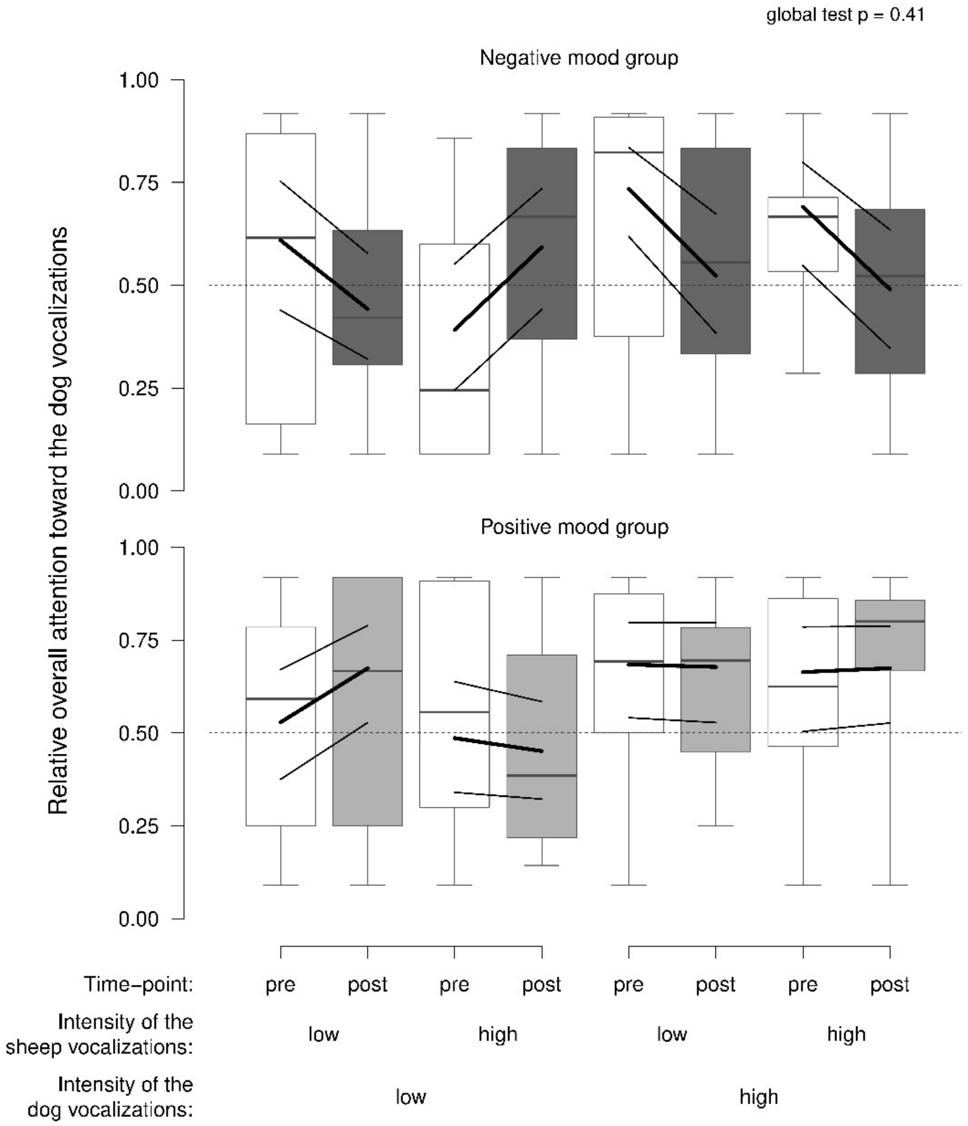

Supplementary Figure S3. Sheep overall attention does not statistically change pre and post mood induction. Relative overall attention directed to the dog vocalizations depending on the intensity of the sheep and dog vocalizations (low, high), the mood group (negative, positive), and the time-point (pre/post mood induction). Statistical information is indicated. Boxplots indicate data range, median, as well as lower and upper quartiles. The white, dark-grey and light-grey boxplots represent the attention pre mood induction, post negative mood induction and post positive mood induction, respectively. Thick black lines are the model estimates, and thin black lines are the 95% confidence intervals of the maximum model (including the main effects and interactions).

Supplementary Table S3. List of the different events used for mood induction. (**a**) Number, type and description of the aversive events (also used in [7], [8], and [9]), duration and evidence of averseness (based on literature and direct observations), (**b**) number and description of the positive events (also used in [20] and [21]), duration and evidence of positivity (based on literature and direct observations), (**c**) schedule of the events (week, day and time of the negative (N1-17) and positive (P1-7) events). All day long (x) corresponds approximately to 9:00-15:30.

(**a**)

N°	Aversive events	Duration	Evidence of averseness
	**Predator signals**		
N1	Aversive contact with a dog (a white Swiss shepherd dog on the leash entered each sheep home pen) [7-9]	3 min / group	Increased heart rate, standing alert [10], stayed away with backward ears
N2	Playback of wolves' howls (credit: U.S. Fish & Wildlife Service; one sequence lasting 4 min) [7]	3 sessions of 4 min	Stayed away, stopped eating
N3	Odor of dog's feces (containers of dog's feces placed in home pens) [7]	All day long	Increased air sniffing, food repellent [11,12]
	**Aversive conspecific signals**		
N4	Odor of blood (containers of sheep's blood collected the same morning in a slaughterhouse and placed in home pens) [7]	All day long	Increased air sniffing [12]
N5	Odor of urine (containers of sheep's urine collected 24h to 48h before in a slaughterhouse and placed in home pens) [7]	All day long	Increased air sniffing [12]
N6	Mixing animals (sheep were mixed between groups, 3 sheep of one pen confronted with 3 more sheep one from three other pens each) [8,9]	120 min	Tried to escape, agonistic interactions, bleating
	**Aversive human signals**		
N7	Individual restraint (all four legs were bound together in home pen) [7,8]	60 min	Increased serum cortisol concentration [8,13], increased breathing rate, tried to escape
N8	Group restraint (all 31 or 16 sheep were confined together in an area outside the home pens measuring l.5 x 1.5 m) [8]	15 min	Tried to escape, defecating, bleating
N9	Isolation (each sheep was led individually outside the home pen with a halter) [9]	3 min / sheep	Defecating/peeing, tried to escape, throwing themselves to the ground
N10<br>N11	Shearing (sheep were shorn) [7]<br>Sham shearing [8,9]	2 min / sheep<br>2 min / sheep	Increased plasma cortisol concentration and stress-induced hyperthermia [14], trembling, stayed away from humans
N12	Presence of a noisy human (loud noises made by a man hitting a bar against metal) [7,8]	15 min	Stayed away with backward ears, stopped eating
N13<br>N14	Disturbance of food access (late food delivery) [7,9]<br>Unreachable food at unpredictable time [7,8]	15 - 90 min<br>15 - 90 min	Scratched and mounted the trough, started feeding immediately once the hay was available

N15	Crossing of a footbath (sheep of one home pen crossed a footbath) [7,8]	5 min / group	Tried to escape, jumped, defecating
N16	Transport (each sheep was individually transported in a box fixed on a trolley) [7-9]	3 min / sheep	Increased heart rate and cortisol concentration [9,10,15], bleating
N17	Unfamiliar object (an umbrella was opened once in front of the all sheep of each home pen)	1 time	Flight response, increased eye white [16-19]

(**b**)

**N°**	**Positive events (description)**	**Duration**	**Evidence of positivity**
P1<br>P2	Objects regularly added (blue ball, big pink ball, tires) [20,21]<br>Foraging substrates (branches, hay net) [20,21]	3 times<br>3 times	Exploration, occupation [21,22], sniffed the object, ate the foraging materials
P3	Gently groomed with a brush or by hand	5 min	Stayed near the stockperson, increased heart rate [23-25], relaxed spine posture, passive or backward ears and semi-closed eyes
P4	A familiar human in green clothes gently handled animals (positive tactile contacts, food reward) [20]	15 min	Stayed calm near the experimenter [25]
P5	Sheep on pasture [21]	All day long	Optimistic-like judgement in horses [21], run to the pasture, anticipated (alert and bleating)
P6	New type of food reward as treats on the passage to and from pasture (apples/carrots, spinach, pasta or butter cakes) [21]	4 times	Sniffed and ate the food
P7	Novel relaxing odor (plastic box containing a compress soaked with essential oil of lavender) [21]	All day long	Sniffed the plastic box, forward ears

(c)

	Week 1							Week 2							Week 3							Week 4						
	M	Tu	W	T	F	Sa	S	M	Tu	W	T	F	Sa	S	M	Tu	W	T	F	Sa	S	M	Tu	W	T	F	Sa	S
N1	12:30											10:00					10:00		10:00					12:30				
N2		09:30		13:30						11:00									13:30				10:00					
N3			x						x								x				x						x	
N4				x								x																
N5					x									x														
N6							13:00													11:00								
N7													10:15								12:00		15:00					
N8		13:30													14:00										10:00			
N9			11:00						10:00								13:00										12:00	
N10								08:15																				
N11																10:00												
N12	09:30					09:30					11:30			15:00				15:15				10:30				11:00		
N13			16:00			08:45						08:15		16:00														
N14	09:45							09:30	17:30		14:40		09:30		12:30	11:00					10:00	10:00				10:30		10:00
N15										12:00																		14:30
N16					14:00																			13:30				
N17				10:15																								
P1																09:15						09:15				09:45		
P2																				09:15		09:15		09:15				
P3															15:30		15:30			09:00	15:30				15:30	09:00		15:30
P4															09:00	09:00	09:00	09:00	09:00	09:00	09:00	09:00	09:00	09:00	09:00	09:00	09:00	09:00
P5															x	x	x	x	x	x	x	x	x	x	x	x	x	x
P6																				16:00	16:00			15:30			15:30	
P7																	x								x			

Supplementary References

1 Praat: doing phonetics by computer v. 6.0.37 (2018).

2 Maigrot, A. L., Hillmann, E., Anne, C. & Briefer, E. F. Vocal expression of emotional valence in Przewalski's horses (Equus przewalskii). *Sci Rep* **7**, 8779, doi:10.1038/s41598-017-09437-1 (2017).

3 de la Torre, M. P., Briefer, E. F., Reader, T. & McElligott, A. G. Acoustic analysis of cattle (Bos taurus) mother–offspring contact calls from a source–filter theory perspective. *Appl Anim Behav Sci* **163**, 58-68 (2015).

4 Naguib, M., Klump, G. M., Hillmann, E., Griessmann, B. & Teige, T. Assessment of auditory distance in a territorial songbird: accurate feat or rule of thumb? *Anim Behav* **59**, 715-721, doi:10.1006/anbe.1999.1354 (2000).

5 Statistical Tools for Measuring Agreement v. 0.8-1 (2015).

6 Linear Mixed-Effects Models using 'Eigen' and S4 v. 1.1-12 (2016).

7 Destrez, A. *et al.* Chronic stress induces pessimistic-like judgment and learning deficits in sheep. *Appl Anim Behav Sci* **148**, 28-36, doi:10.1016/j.applanim.2013.07.016 (2013).

8 Doyle, R. E. *et al.* Measuring judgement bias and emotional reactivity in sheep following long-term exposure to unpredictable and aversive events. *Physiol Behav* **102**, 503-510, doi:10.1016/j.physbeh.2011.01.001 (2011).

9 Coulon, M. *et al.* Effects of prenatal stress and emotional reactivity of the mother on emotional and cognitive abilities in lambs. *Dev Psychobiol* **57**, 626-636, doi:10.1002/dev.21320 (2015).

10 Baldock, N. M. & Sibly, R. M. Effects of handling and transportation on the heart rate and behaviour of sheep. *Appl Anim Behav Sci* **28**, 15-39 (1990).

11 Arnould, C., Malosse, C., Signoret, J.-P. & Descoin, C. Which chemical constituents from dog feces are involved in its food repellent effect in sheep? *Journal of Chemical Ecology* **24** (1998).

12 Terlouw, E. M. C., Boissy, A. & Blinet, P. Behavioural responses of cattle to the odours of blood and urine from conspecifics and to the odour of faeces from carnivores. *Appl Anim Behav Sci* **57**, 9-21 (1998).

13 Doyle, R. E., Fisher, A. D., Hinch, G. N., Boissy, A. & Lee, C. Release from restraint generates a positive judgement bias in sheep. *Appl Anim Behav Sci* **122**, 28-34, doi:10.1016/j.applanim.2009.11.003 (2010).

14 Sanger, M. E., Doyle, R. E., Hinch, G. N. & Lee, C. Sheep exhibit a positive judgement bias and stress-induced hyperthermia following shearing. *Appl Anim Behav Sci* **131**, 94-103, doi:10.1016/j.applanim.2011.02.001 (2011).

15 Schwartzkopf-Genswein, K. S. *et al.* Effects of pre-haul management and transport duration on beef calf performance and welfare. *Appl Anim Behav Sci* **108**, 12-30, doi:10.1016/j.applanim.2006.11.012 (2007).

16 Sandem, A. I., Janczak, A. M. & Braastad, B. O. A short note on effects of exposure to a novel stimulus (umbrella) on behaviour and percentage of eye-white in cows. *Appl Anim Behav Sci* **89**, 309–314, doi:10.1016/j.applanim.2004.06.011 (2004).

17 Reefmann, N., Muehlemann, T., Wechsler, B. & Gygax, L. Housing induced mood modulates reactions to emotional stimuli in sheep. *Appl Anim Behav Sci* **136**, 146-155, doi:10.1016/j.applanim.2011.12.007 (2012).

18 Austin, N. P. & Rogers, L. J. Asymmetry of flight and escape turning responses in horses. *Laterality* **12**, 464-474, doi:10.1080/13576500701495307 (2007).

19 Robins, A. & Phillips, C. Lateralised visual processing in domestic cattle herds responding to novel and familiar stimuli. *Laterality* **15**, 514-534, doi:10.1080/13576500903049324 (2010).

20 Destrez, A., Deiss, V., Leterrier, C., Calandreau, L. & Boissy, A. Repeated exposure to positive events induces optimistic-like judgment and enhances fearfulness in chronically stressed sheep. *Appl Anim Behav Sci* **154**, 30-38, doi:10.1016/j.applanim.2014.01.005 (2014).

21 Lansade, L. *et al.* Behavioral and Transcriptomic Fingerprints of an Enriched Environment in Horses (Equus caballus). *PLoS One* **9**, e114384, doi:10.1371/journal.pone.0114384 (2014).

22 Burman, O. H., Parker, R. M., Paul, E. S. & Mendl, M. A spatial judgement task to determine background emotional state in laboratory rats, Rattus norvegicus. *Anim Behav* **76**, 801-809, doi:10.1016/j.anbehav.2008.02.014 (2008).

23 Baciadonna, L., Nawroth, C. & McElligott, A. G. Judgement bias in goats (Capra hircus) investigating the effects of human grooming. *Peer J* **4**, e2485, doi:10.7717/peerj.2485 (2016).
24 Reefmann, N., Wechsler, B. & Gygax, L. Behavioural and physiological assessment of positive and negative emotion in sheep. *Anim Behav* **78**, 651–659, doi:10.1016/j.anbehav.2009.06.015 (2009).
25 Tallet, C., Veissier, I. & Boissy, A. Human contact and feeding as rewards for the lamb's affinity to their stockperson. *Appl Anim Behav Sci* **94**, 59–73, doi:10.1016/j.applanim.2005.02.007 (2005).
